# Supplementary material for: Management of Synchronous Extrathoracic Oligometastatic Non-Small Cell Lung Cancer
Source: Cancers (Basel). 2021 Apr 15;13(8):1893. doi: 10.3390/cancers13081893 (PMC8071146; doi:10.3390/cancers13081893)
Supplement: Supplementary file 1 [file cancers-13-01893-s001.zip › cancers-1157944-supplementary-for xml/cancers-1157944-supplementary- for proof.docx]

Management of Synchronous Extrathoracic Oligometastatic Non-Small Cell Lung Cancer

Gregory D. Jones, Harry B. Lengel, Meier Hsu, Kay See Tan, Raul Caso, Amanda Ghanie, James G. Connolly,
Manjit S. Bains, Valerie W. Rusch, James Huang, Bernard J. Park, Daniel R. Gomez, David R. Jones and
Gaetano Rocco


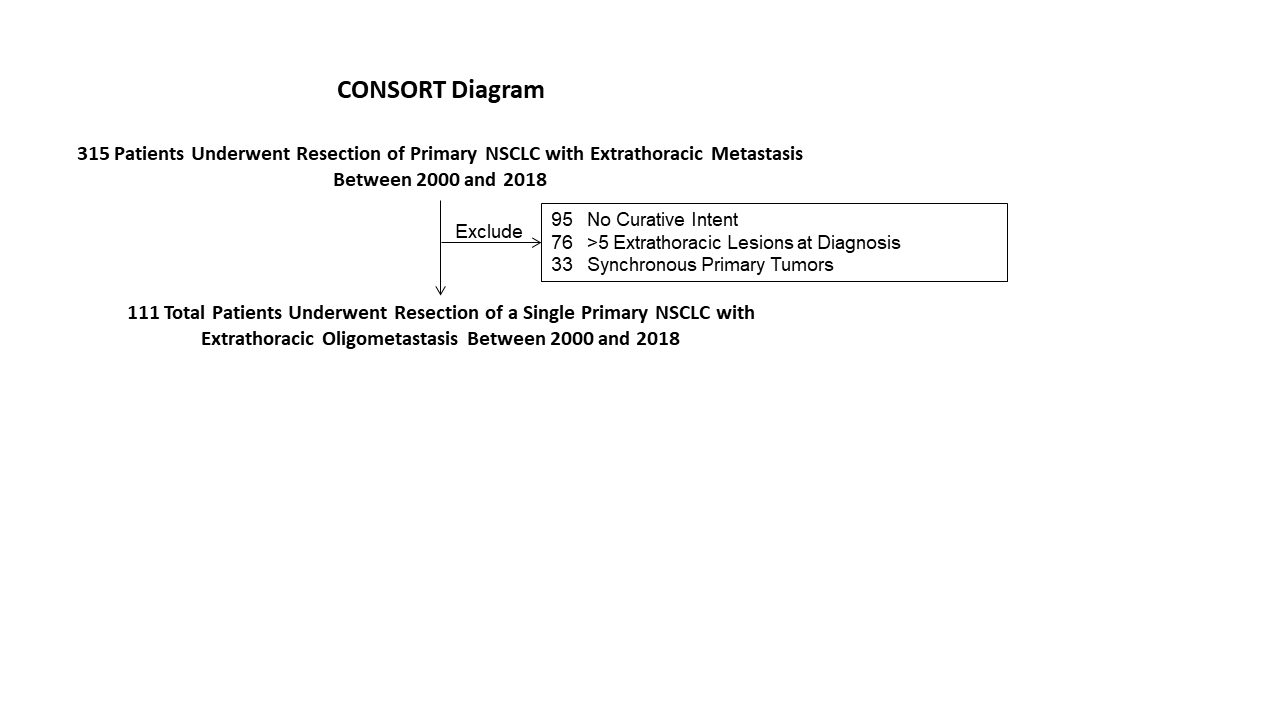


**Figure S1.** CONSORT diagram.


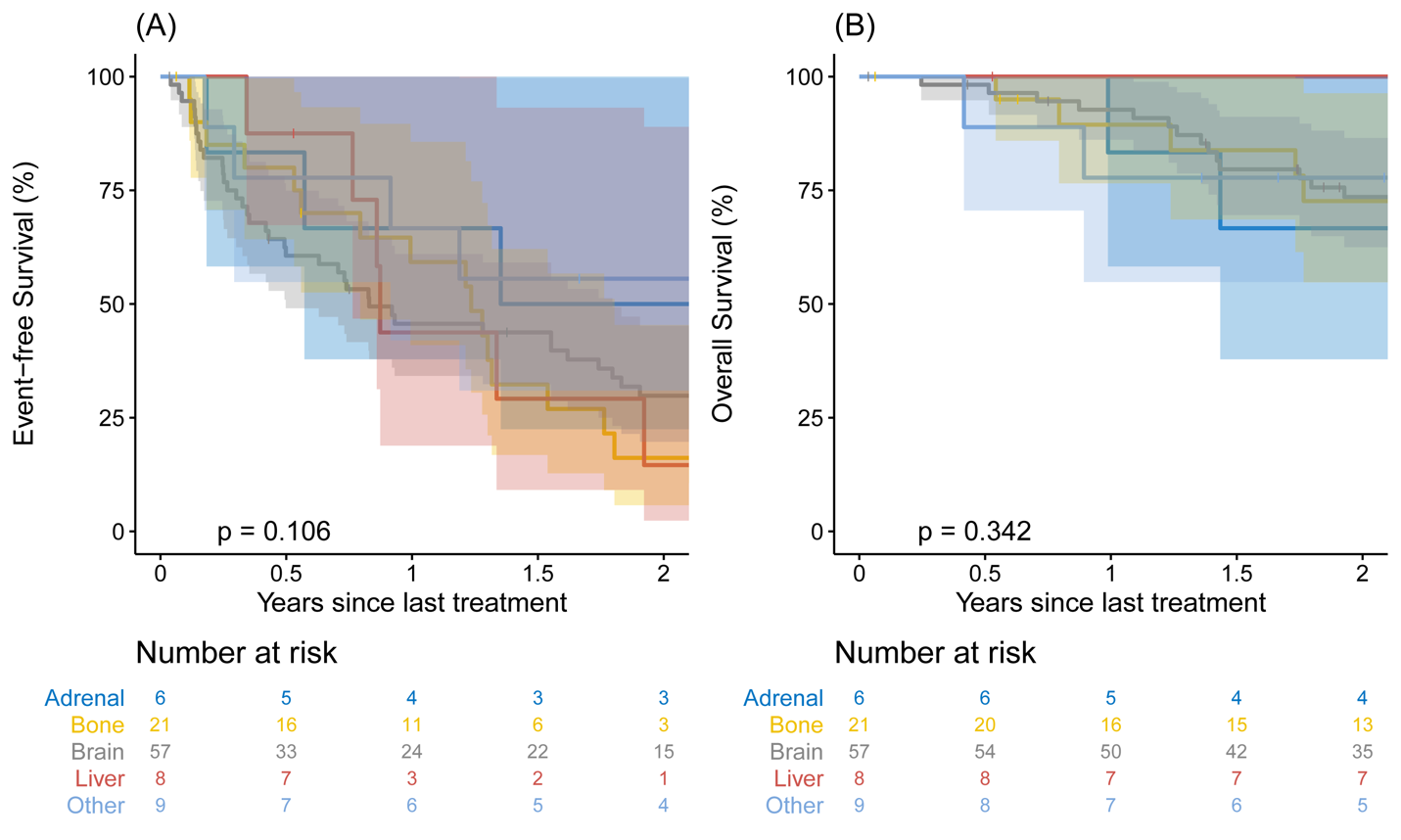


**Figure S2.** Kaplan-Meier 3-year event-free survival (A) and overall survival (B) estimates by metastatic site at diagnosis: adrenal, bone, brain, and other. “Other” includes eye, intestine, omentum, pancreas, scalp, spleen, and perianal soft tissue.


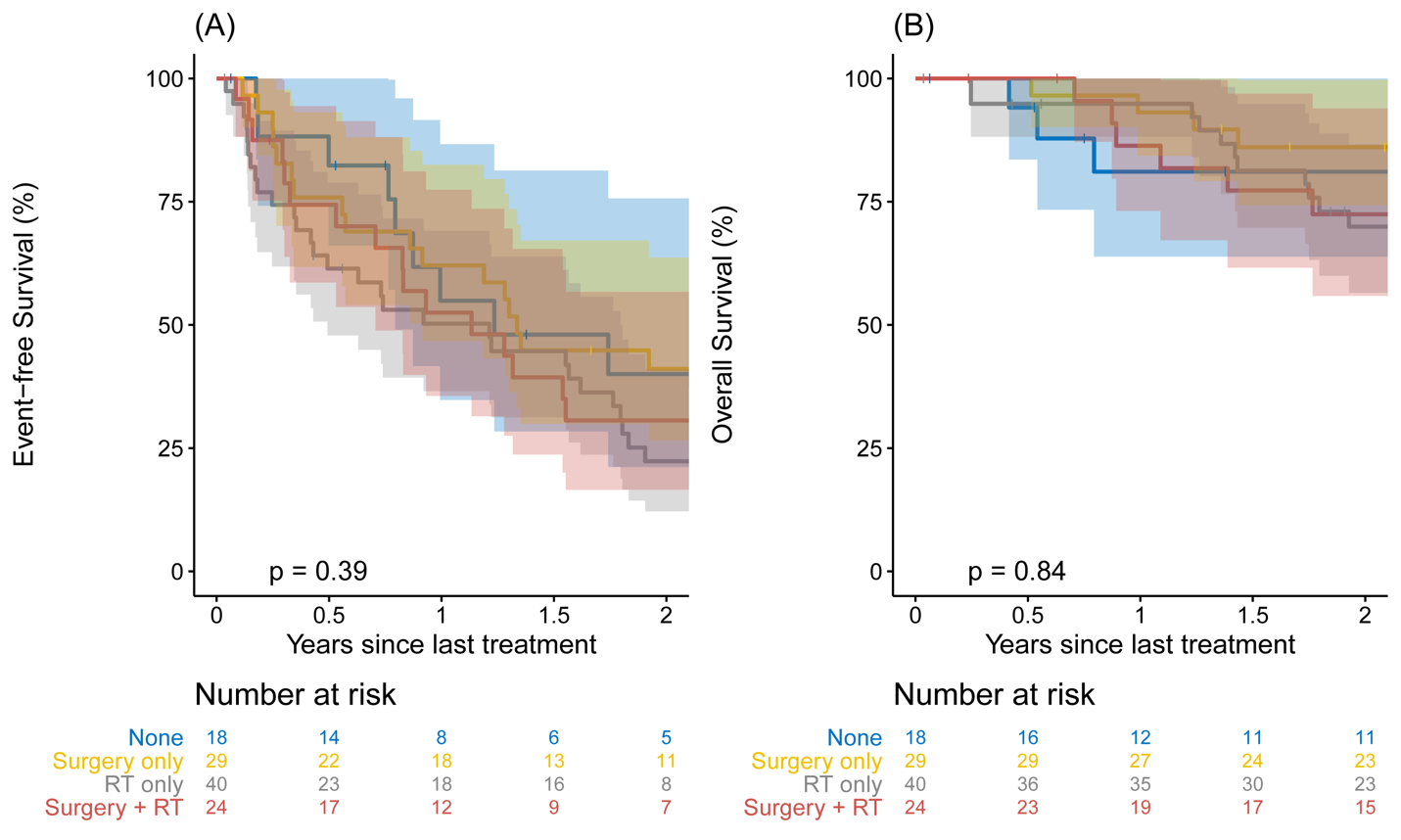


**Figure S3.** Kaplan-Meier 3-year event-free survival (A) and overall survival estimates (B) by type of metastatic local consolidative therapy received: none, surgery only, radiation therapy (RT) only, and surgery + RT.


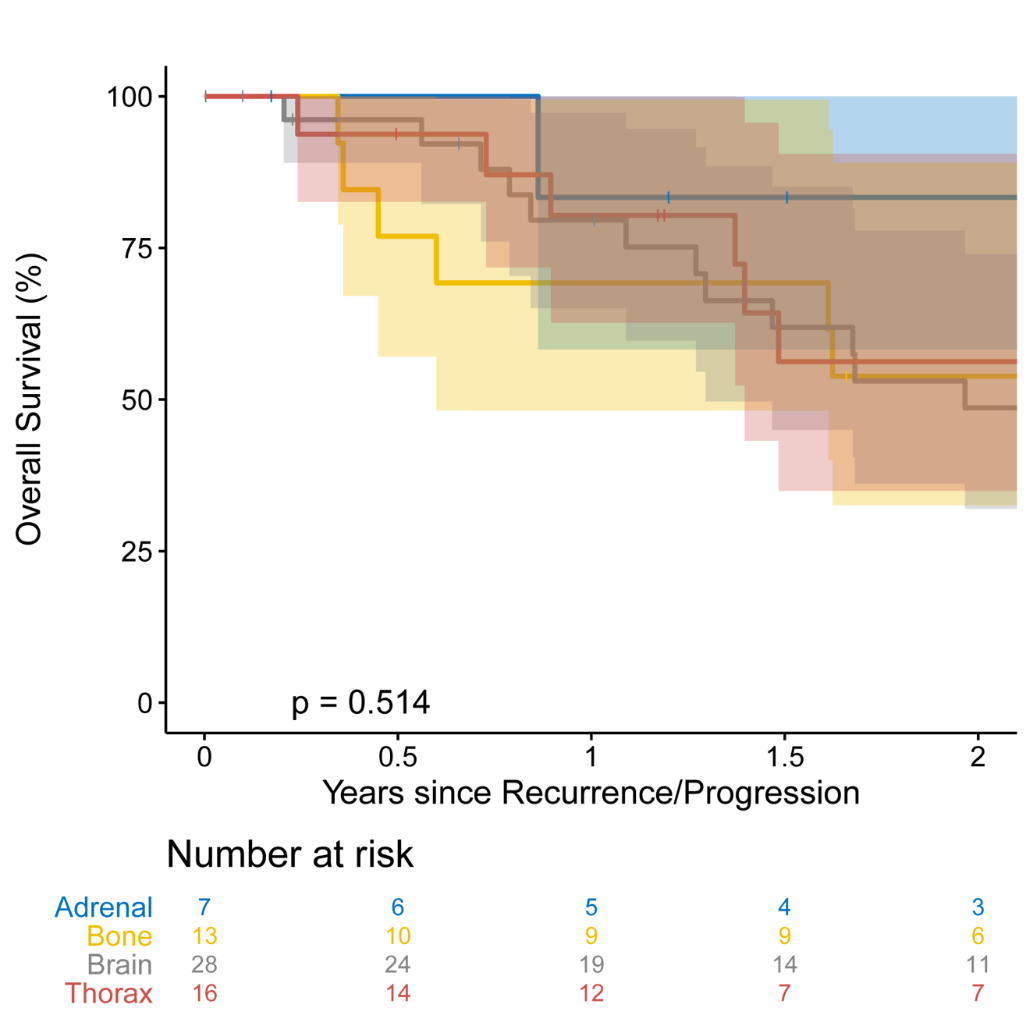


**Figure S4.** Kaplan-Meier 3-year postrecurrence survival estimates by site (adrenal, bone, brain, or thorax) of recurrence or progression (*n* = 77). The Y-axis represents the time to death from the date of first recurrence or progression. RT, radiation therapy.

**Table S1.** Complete univariable analysis for factors associated with event-free survival.

| **Variable** | **HR** | **95% CI** | ***P*** |
| --- | --- | --- | --- |
| Age at surgery, years | 0.99 | (0.97-1.01) | 0.2 |
| FEV1, % (n=103) | 1.00 | (0.98-1.01) | 0.9 |
| DLCO, % (n=103) | 1.00 | (0.99-1.01) | 0.5 |
| Induction therapy | 0.92 | (0.56-1.52) | 0.7 |
| Metastasis location at diagnosis |  |  |  |
| Bone | Ref | - | - |
| Adrenal | 0.32 | (0.09-1.11) | 0.074 |
| Brain | 0.75 | (0.43-1.31) | 0.3 |
| Liver | 0.88 | (0.37-2.11) | 0.8 |
| Multiple | 0.29 | (0.11-0.80) | 0.02 |
| Other | 0.53 | (0.22-1.30) | 0.2 |
| Total metastatic lesions | 0.72 | (0.49-1.05) | 0.085 |
| Metastasis treated | 0.55 | (0.18-1.19) | 0.3 |
| Histologic subtype |  |  |  |
| Lepidic/acinar/papillary | Ref | - | - |
| Micropapillary/solid | 2.11 | (1.02-4.36) | 0.043 |
| Final pathologic diagnosis |  |  |  |
| Adenocarcinoma | Ref | - | - |
| Squamous cell carcinoma | 0.76 | (0.27-2.15) | 0.6 |
| Other NSCLC | 0.82 | (0.44-1.52) | 0.5 |
| No viable tumor | 0.45 | (0.16-1.23) | 0.12 |
| Lymphovascular invasion | 1.85 | (1.16-2.96) | 0.010 |
| Visceral pleural invasion | 1.58 | (1.01-2.46) | 0.045 |
| Pathologic tumor size, cm | 1.15 | (1.03-1.28) | 0.013 |
| Pathologic N stage |  |  |  |
| 0 | Ref | - | - |
| 1/2 | 1.57 | (1.01-2.45) | 0.047 |
| Pathologic M stage |  |  |  |
| 0 | Ref | - | - |
| 1a | 1.18 | (0.32-4.37) | 0.8 |
| 1b/1c | 1.74 | (0.87-3.48) | 0.12 |
| Pathologic stage (AJCC 8) |  |  |  |
| 0 | Ref | - | - |
| I | 2.08 | (0.38-11.4) | 0.4 |
| II/III | 2.70 | (0.45-16.2) | 0.3 |
| IV | 3.04 | (0.75-12.4) | 0.12 |
| Extent of resection |  |  |  |
| R0 | Ref | - | - |
| R1/R2 | 1.64 | (0.59-4.54) | 0.3 |

AJCC, American Joint Committee on Cancer; DLCO, diffusion capacity of the lungs for carbon monoxide; FEV1, forced expiratory volume in 1 second; NSCLC, non-small cell lung cancer; ref, reference; SUVmax, maximum standardized uptake value; VATS, video-assisted thoracoscopic surgery.

**Table S2.** Complete univariable analysis for factors associated with overall survival.

| **Variable** | **HR** | **95% CI** | ***P*** |
| --- | --- | --- | --- |
| Age at surgery, years | 1.00 | (0.98-1.02) | 0.9 |
| FEV1, % (n=103) | 0.99 | (0.98-1.01) | 0.6 |
| DLCO, % (n=103) | 1.00 | (0.99-1.02) | 0.6 |
| Induction therapy | 0.52 | (0.30-0.89) | 0.018 |
| Metastasis location at diagnosis |  |  |  |
| Bone | Ref | - | - |
| Adrenal | 0.51 | (0.14-1.81) | 0.3 |
| Brain | 0.60 | (0.31-1.14) | 0.12 |
| Liver | 0.57 | (0.19-1.73) | 0.3 |
| Multiple | 0.27 | (0.08-0.95) | 0.042 |
| Other | 0.48 | (0.14-1.70) | 0.3 |
| Total metastatic lesions | 0.51 | (0.27-1.37) | 0.10 |
| Metastasis treated | 0.65 | (0.37-1.12) | 0.2 |
| Histologic subtype |  |  |  |
| Lepidic/acinar/papillary | Ref | - | - |
| Micropapillary/solid | 1.97 | (0.75-5.17) | 0.2 |
| Final pathologic diagnosis |  |  |  |
| Adenocarcinoma | Ref | - | - |
| Squamous cell carcinoma | 1.39 | (0.48-4.08) | 0.5 |
| Other NSCLC | 1.00 | (0.49-2.05) | 0.9 |
| No viable tumor | 0.23 | (0.03-1.64) | 0.14 |
| Lymphovascular invasion | 1.77 | (1.01-3.10) | 0.047 |
| Visceral pleural invasion | 2.45 | (1.42-4.21) | 0.001 |
| Pathologic tumor size, cm | 1.21 | (1.06-1.38) | 0.004 |
| Pathologic N stage |  |  |  |
| 0 | Ref | - | - |
| 1/2 | 2.05 | (1.18-3.56) | 0.010 |
| Pathologic M stage |  |  |  |
| 0 | Ref | - | - |
| 1a | 0.96 | (0.20-4.62) | 0.9 |
| 1b/1c | 1.35 | (0.61-2.97) | 0.5 |
| Pathologic stage (AJCC 8) |  |  |  |
| 0 | Ref | - | - |
| I | 1.65 | (0.17-16.0) | 0.7 |
| II/III | 1.99 | (0.20-19.4) | 0.6 |
| IV | 2.14 | (0.29-15.6) | 0.5 |
| Extent of resection |  |  |  |
| R0 | Ref | - | - |
| R1/R2 | 1.92 | (0.59-6.17) | 0.3 |
| Adjuvant therapy | 0.91 | (0.49-1.67) | 0.9 |

AJCC, American Joint Committee on Cancer; DLCO, diffusion capacity of the lungs for carbon monoxide; FEV1, forced expiratory volume in 1 second; NSCLC, non-small cell lung cancer; ref, reference; SUVmax, maximum standardized uptake value; VATS, video assisted thoracoscopic surgery.

**Table S3.** Patient selection criteria in oligometastatic non-small cell lung cancer.

| **Criteria** | **Literature criteria for surgery of the lung primary as LCT** | **Current MSKCC study** |
| --- | --- | --- |
| ECOG Performance Status | 0-1 | 0-1 |
| Cardiopulmonary reserve | Permissive^28^ | Permissive |
| Number of metastases | 1-3^26,28^ | Not a factor -  All ≤ 5 metastatic lesions |
| Nodal involvement | N0^25,28-30^ | N0 |
| Anticipated completeness of resection (R0) | Yes^28^ | Yes |
| Disease Free Interval (DFI) | >6-12 months^29^ | --- |
| Site of metastases | Adrenal, Brain, Lung^30^ | All extrathoracic sites^a^ |
| Timing of metastases | Metachronous^25,30^ | Synchronous |

LCT = local consolidative therapy. MSKCC = Memorial Sloan Kettering Cancer Center. ECOG = Eastern Cooperative Oncology Group. ^a^Extrathoracic metastatic sites included: adrenal, brain, bone, liver, eye, intestine, omentum, pancreas, scalp, spleen, and perianal soft tissue.

**Video S1.** Our findings regarding the role of surgical resection of the primary tumor in select patients with oligometastatic non-small cell lung cancer.
